# Periostin Is a Candidate Regulator of the Host Microenvironment in Regeneration of Pulp and Dentin Complex and Periodontal Ligament in Transplantation with Stem Cell-Conditioned Medium

**DOI:** 10.1155/2024/7685280

**Published:** 2024-02-23

**Authors:** Shintarou Sakatoku, Yuki Hayashi, Taku Futenma, Yoshihiko Sugita, Ryo Ishizaka, Hiroyuki Nawa, Koichiro Iohara

**Affiliations:** ^1^Department of Pediatric Dentistry, School of Dentistry, Aichi-Gakuin University, Suemoridouri 2-11, Chikusa-ku, Nagoya 464-8651, Aichi, Japan; ^2^Department of Oral Pathology and Forensic Odontology, School of Dentistry, Aichi Gakuin University, 1-1-100 Kusumoto-cho, Chikusa-ku, Nagoya 464-8650, Aichi, Japan; ^3^Department of Dental Regenerative Medicine, Center of Advanced Medicine for Dental and Oral Diseases, National Center for Geriatrics and Gerontology, Research Institute, Morioka 7-430, Obu 474-8511, Aichi, Japan

## Abstract

**Purpose:**

The microenvironment is required for tissues to maintain their properties *in vivo*. This microenvironment encompasses the types and three-dimensional arrangement of cells forming the tissues, and their interactions with neighboring cells and extracellular matrices, as represented by the stem cell niche. Tissue regeneration depends not on the original tissue source of the transplanted cells, but on the microenvironment in which they are transplanted. We have previously reported pulp regeneration in a heterotopic root graft model by transplantation of conditioned medium alone, which suggests that host-derived cells expressing receptors for migration factors in conditioned medium migrate into the root canal and cause pulp regeneration. Regenerative medicine is needed to restore the original function of complex tissues. To achieve this, it is necessary to reproduce the changes in the microenvironment of the host tissue that accompany the regenerative response. Therefore, it is important to reproduce the microenvironment *in vivo* for further development of tissue regeneration therapy. Periostin is also found in the epithelial–mesenchymal junction, with expression sites that differ depending on the mineralized matrix stage, and is involved in regulation of calcification.

**Methods:**

We investigate whether periostin contributes to microenvironmental changes in regenerated pulp tissue. Dental pulp stem cells were induced into dentin, and gene expression of DSPP, nestin, DMP1, Runx2, and periostin was analyzed by qPCR and protein expression by IHC. Similarly, gene expression was analyzed using qPCR and protein expression using IHC in regenerated dental pulp obtained by ectopic transplantation.

**Results:**

Since these regenerated tissues were observable on the same slice, it was possible to understand changes in the microenvironment within the tissues.

**Conclusions:**

Periostin promoted proliferation of pulp stem cells, migration in type I collagen, and calcification in regenerated pulp, which strongly suggests that periostin is a promising candidate as a factor that contributes to the microenvironment of regenerated pulp.

## 1. Introduction

The microenvironment is the environment necessary for a tissue to maintain its properties *in vivo*, encompassing the types of cells that make up the tissue, their three-dimensional arrangement, and their interactions through neighboring cells and the extracellular matrix, as exemplified by the stem cell niche [1, 2]. Stem cell fate determination is controlled by the microenvironment [3], which indicates that tissue regeneration and changes in host tissues depend on the microenvironment of the transplanted cells, as well as on the original tissue source of these cells [4, 5]. Therefore, it is important to understand and reproduce the microenvironment *in vivo* for further development of tissue regeneration therapy [6, 7].

Dental trauma is a common disorder in clinical practice in pediatric dentistry. Trauma occurs extensively to the tooth, pulp, periodontal tissue, and alveolar bone, and particular characteristics in childhood must also be taken into account, including the relationship with the subsequent permanent tooth and the amount of root formation in young permanent teeth. Trauma treatment is needed using regenerative medicine to restore the original function of complex tissues, rather than just repairing the pulp–dentin complex, periodontal ligament, and alveolar bone. This requires an understanding and reproduction of changes in the microenvironment of the host tissue during the regenerative reaction.

Regeneration is viewed as a process of replication and rebuilding of some aspects of tissue development [8]. Periostin is an adhesion molecule in preosteoblasts, an extracellular matrix protein in the lung, heart, intestinal tract, skin, and periodontal ligament tissues at steady state, and a regulator of collagen fiber formation. It is expressed in many tissues, especially in the fetal stage, and is involved in cell migration and survival, suggesting its contribution to developmental stages [9]. In tooth development periostin is also found in the epithelial–mesenchymal junction, with expression sites that differ depending on the mineralized matrix stage, and is involved in regulation of calcification. However, possible roles of periostin in dental pulp stem cells have not been determined. Therefore, in this study, we analyzed the chronological dynamics of periostin in induction of stem cell differentiation.

In addition to the pulp–dentin complex, regeneration of the periodontal ligament is essential for application of regenerative medicine in trauma treatment. Direct involvement *in situ* should also be taken into account. Therefore, models for both *in vitro* and *in vivo* evaluation are required. Maintenance of the microenvironment of tooth enamel and dentin after extraction and removal from the body can be achieved using stem cells and cell sheets in heterotopic transplant [10, 11], and this approach can be used as a model for evaluating pulp and periodontal ligament regeneration [12, 13]. The secretome, which is the chemical microenvironment of dental pulp stem cells, can be extracted from stem cell-conditioned medium in which the cells accumulate [14]. We have previously reported pulp regeneration in a heterotopic root graft model by transplantation of conditioned medium alone, which suggests that host-derived cells expressing receptors for migration factors in conditioned medium migrate into the root canal and cause pulp regeneration. Therefore, a supernatant transplantation model for heterotopic root canal transplantation can be used to evaluate changes in the host tissue microenvironment in the regeneration response. However, we did not previously examine regeneration of periodontal ligament on the external surface of the tooth root, which is similarly preserved. Therefore, in the current study, we evaluated simultaneous regeneration of pulp and periodontal ligament in a heterotopic root graft, and we conducted an immunohistological analysis of posttransplantation tissues on Days 7, 14, and 21 to examine whether periostin contributes to microenvironmental changes in regenerated pulp tissues.

## 2. Materials and Methods

### 2.1. Fractionation of Human Dental Pulp Stem Cells and Preparation of Human Dental Pulp Stem Cell-Conditioned Medium

Human dental pulp stem cells (hDPSCs) were obtained from dental pulp collected from permanent teeth of different patients (18–34 years old) without systemic disease who underwent tooth extraction at the Department of Pediatric Dentistry, School of Dentistry, Aichi Gakuin University, using enzymatic digestion based on the procedure of Hayashi et al. [15]. First, the dental pulp was shredded and 0.2% collagenase (Wako Pure Chemical, Osaka, Japan) was added to the pulp to disperse the cells. After three passages, the fractionated hDPSCs were switched to serum-free medium at 70% confluency and collected as conditioned medium (CM) after 24 hr. The collected supernatant was concentrated to 500 *μ*g/mL using Amicon Ultra-15 Centrifugal Filters Ultracel®-3K (Merck Millipore, USA) and incubated with a protease inhibitor (Halt™ Protease Inhibitor Single-Use Cocktail, Thermo Fisher Scientific, Rockford, IL, USA). The protein concentration in CM was determined with a Qubit® Assay kit (Thermo Fisher Scientific, Tokyo, Japan). Cell collection was performed with the approval of the Ethics Committee of the School of Dentistry, Aichi Gakuin University (approval no.: 639). Experiments to be performed after this were performed using cells derived from different individuals.

### 2.2. Immunohistological and PCR Analyses of Dentin Induction

hDPSCs after three passages (5 × 10^4^ cells) were seeded onto each well of a 24-well plate in equal volume and cultured to 100% confluency. The cells were then cultured for 21 days in medium containing 50 *μ*L/mL ascorbic acid, 4 mol/L Pi, 10 *μ*g/mL BMP-2, and 10% FBS (Dulbecco's Modified Eagle Medium, DMEM) as dentin induction medium. As a comparison group, hDPSC CM (final concentration: 10 *μ*g/mL) was added to the dentin induction medium. The medium was replaced every 3 days. Immunostaining was performed using mouse anti-DSPP antibody (Santa Cruz, sc-73632), mouse anti-nestin antibody (GeneTex, GT623), rabbit anti-DMP1 antibody (Affinity Biosciences, DF8825), anti-Runx2 antibody (Proteintech, 20700-11AP), and anti-periostin antibody (abcam, ab14041), 7, 14, and 21 days after dentin induction. Nuclear staining with Hoechst 33342 (Sigma–Aldrich, St Louis, MO, USA) was observed using a CKX53 fluorescence microscope (Olympus, Tokyo, Japan).

Pulp stem cells induced under the same conditions for 7, 14, and 21 days were collected and RNA was immediately isolated by a pressurized method using a QuickGene RNA cultured cell kit S (Kurabo, Tokyo, Japan). RNA isolation was performed in the following steps. Lysates were prepared from collected tissues and pressurized to isolate RNA using a QuickGene RNA tissue kit S II (Kurabo). gDNA was removed by DNase treatment (Qiagen, Tokyo, Japan), and the isolated total RNA solution was stored at −80°C. qPCR was performed with the collected RNA using a fast real-time PCR system (GF-Q150, Kurabo) to analyze *DSPP*, *nestin*, *DMP1*, *Runx2*, and p*eriostin* mRNA expression. First, 2 *μ*L of isolated total RNA, 5 *μ*L of fluorescent reagent containing reverse transcriptase (Quick One-step RT-PCR Master Mix, Kurabo), and 3 *μ*L of primer for the genes (*DSPP*, *nestin*, *DMP1*, *Runx2*, and p*eriostin*) were combined to give a total reagent volume of 10 *μ*L. After confirming that there were no bubbles present, a Quick PCR Chip was added and fast real-time PCR was performed. *Ct* values obtained after 45 cycles were compared with those for the housekeeping gene *β*-actin (*ΔCt*). Relative expression levels of target mRNAs were obtained by the *ΔΔCt* method as percentages of *ΔCt* of the control (Table 1).

### 2.3. Murine Heterotopic Root Graft Modeling

Heterotopic root graft models were used to examine the tissue regenerative capacity of CM based on the method of Hayashi et al. [15]. The root canal of a human permanent tooth cut to 6 mm in width was enlarged to 2 mm in diameter, and remaining pulp and periodontal ligaments were removed. One end of the root canal was closed with hydraulic cement, and CM (prepared in Section 1) was mixed with collagen TE (Nitta Gelatin, Tokyo, Japan) to a final concentration of 10 *μ*g/mL and injected into the root canal to make a graft. The CM inside the graft was derived from other individuals. Four grafts each were made and implanted into the abdominal cavity of 5-week-old SCID mice. The respective grafts were collected 3, 7, 14, and 21 days after implantation, fixed in paraformaldehyde (Nacalai Tesque, Kyoto, Japan), and demineralized in Kalkitox (Wako, Osaka, Japan) at 4°C for 1 week. Longitudinal paraffin sections of 5 *μ*m were used as grafts (*n* = 4). All animal experiments were conducted in accordance with the Rules for the Conduct of Animal Experiments of the School of Dentistry, Aichi Gakuin University (approval no.: AGUD487).

### 2.4. Tissue Regeneration Analysis

Grafts were stained with hematoxylin–eosin (HE) and immunostained with rabbit anti-periostin antibody (abcam, ab14041). The regenerated pulp volume and regenerated periodontal ligament in grafts were visualized using an optical microscope BX53 (Olympus). The internal area of each 4-lot, supernatant-implanted root canal was analyzed using cellSens imaging software (Olympus) to estimate the ratio of regenerated pulp area to root canal area and calculate the mean. The width of regenerated periodontal ligament was measured at six random points per graft and the mean was also calculated.

### 2.5. Immunohistological Analysis of Calcification Regulators in Regenerated Tissues

Graft slices were immunostained with mouse anti-DSPP, anti-nestin (GeneTax, LA, USA, GT623) and rabbit anti-periostin antibodies to evaluate *in situ* expression and localization of calcification regulators in regenerated dental pulp and periodontal ligament. After antibody staining, nuclear staining was performed with Hoechst 33342. Localization of *in situ* expression was evaluated 7, 14, and 21 days after heterotopic transplantation. The number of antibody-expressing cells in photographed tissues was determined using ImageJ software (National Institute of Health, USA) and the positive rate was calculated from the total number of cells.

### 2.6. PCR Analysis of Calcification Regulators in Regenerated Dental Pulp Tissues

To analyze expression of *DSPP*, *nestin*, *DMP1*, *Runx2*, and *periostin* in regenerated pulp tissues, tissues inside the tooth root were collected 7, 14, and 21 days after heterotopic root grafting and RNA was isolated immediately. RNA isolation was performed by a nucleic acid isolation system using a pressurized method with a QuickGene RNA tissue kit S II (Kurabo, Tokyo, Japan) with the same procedures as those in Section 2. The isolated total RNA solution was stored at −80°C. qPCR was performed with the collected RNA using a fast real-time PCR system to analyze *DSPP*, *nestin*, *DMP1*, *Runx2*, and *periostin* mRNA expression. *Ct* values of samples were compared with those for the housekeeping gene *β*-actin (*ΔCt*). Relative expression levels of target mRNAs were obtained by the *ΔΔCt* method as percentages of *ΔCt* for the control sample (Table 2).

### 2.7. Analysis of Promotion of Cell Proliferation by Periostin

Promotion of cell proliferation by periostin in pulp stem cells was analyzed in 5 × 10^4^ hDPSCs seeded in 24-well plates in the same volume in each well. Recombinant periostin protein (100 ng/mL; Funakoshi, Tokyo, Japan) or CM (final concentration: 10 *μ*g/mL) was added, and after 6, 12, and 24 hr, a cell counting kit-8 (Dojin Chemical Research Institute, Kumamoto, Japan) was used to determine absorbance at 450 nm using a plate reader SPARK10M® (Tecan Japan, Kanagawa, Japan).

### 2.8. Analysis of Promotion of Cell Hypotaxis by Periostin

Promotion of cell haptotaxis by periostin in dental pulp stem cells was analyzed using Haptotaxis CytoSelect® (Cell Biolabs Inc, Tokyo, Japan). First, 5 × 10^5^ hDPSCs were mixed with serum-free medium containing 100 ng/mL recombinant periostin (Funakoshi) or CM (final concentration: 10 *μ*g/mL) and seeded in 24-well plates coated with 8-*μ*m pore polycarbonate membranes. After 24 hr, migrating cells that had passed through the type I collagen-coated membrane and adhered to the lower part of the membrane were reacted with cell detachment solution for 10 min and measured by plate reader fluorescence (520 nm).

### 2.9. Statistical Analysis

All data obtained were from cells and cell supernatants derived from different individuals and were performed three times each. Data are expressed as mean ± standard deviation. The sample size and number of *n* for each experiment were four. Statistical analysis was performed by one-way analysis of variance (ANOVA) followed by Tukey's test, using SPSS ver.27.0.1® (IBM, NY, USA).

## 3. Results and Discussion

### 3.1. Results

#### 3.1.1. Expression of DSPP, Nestin, DMP1, Runx2, and Periostin in Dental Pulp Stem Cells during Dentin Induction

**Figure 1:**
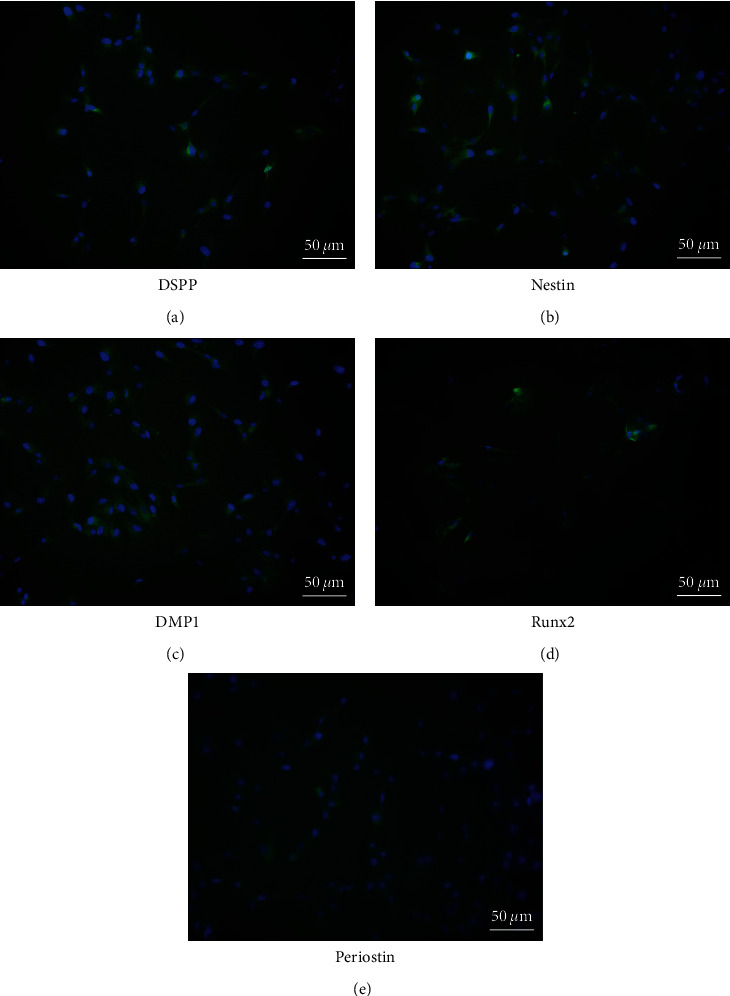
Expression of *DSPP*, *nestin*, *DMP1*, *Runx2*, *and periostin* by dentin induction of dental pulp stem cells. Immunohistochemical staining images after the start of dentin induction 21 days. (Bule: Hoechst 33342 and Green: (a) DSPP, (b) nestin, (c) DMP1, (d) Runx2, and (e) periostin).

**Figure 2:**
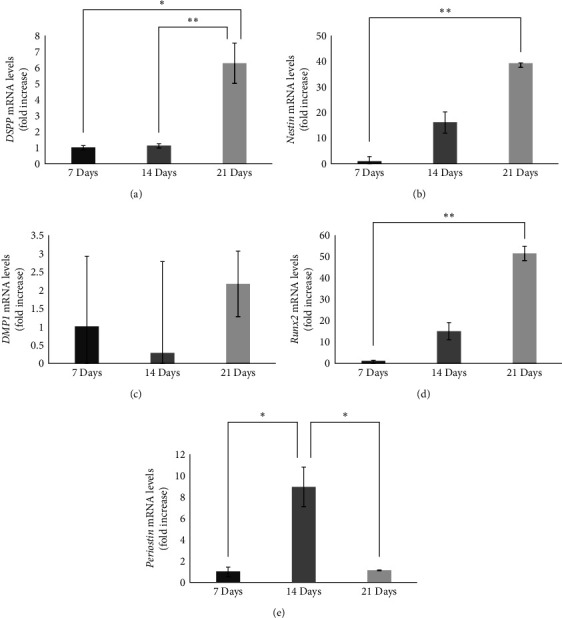
Gene expression of *DSPP*, *nestin*, *DMP1*, *Runx2*, and *periostin* by dentin induction of dental pulp stem cells. Relative gene expression levels 7, 14, and 21 days after dentin induction was initiated. (Relative quantification by *ΔΔCt* method after qPCR analysis; (a) *DSPP*, (b) *nestin*, (c) *DMP1*, (d) *Runx2*, and (e) *periostin*). Mean ± standard error with *n* = 4,  ^*∗*^*P* < 0.05 and  ^*∗∗*^*P* < 0.01.

Immunohistochemical staining for DSPP, nestin, DMP1, Runx2, and periostin, was performed in pulp stem cells cultured in dentin-induced medium. On Day 21 of induction, DSPP, nestin, DMP1, Runx2, and periostin-positive cells were observed in the cytoplasm (Figure 1(a)–1(e)). RNA was isolated from the pulp stem cells cultured in dentin-induced medium, and qPCR analysis was performed. The results showed no significant difference in expression of *DSPP* on Days 7 and 14 of dentin induction, but expression on Day 21 was 6.2 times higher than that on Day 7 (Figure 2(a)). No significant difference in expression of n*estin* on Days 7 and 14 of dentin induction, but expression on Day 21 was 38.5 times higher than that on Day 7 (Figure 2(b)). No significant difference in expression of *DMP1* on Days 7, 14, and 21 of dentin induction (Figure 2(c)). No significant difference in expression of *Runx2* on Days 7 and 14 of dentin induction, but expression on Day 21 was 51.3 times higher than that on Day 7 (Figure 2(d)).

In a similar analysis of expression of *periostin*, the expression level was 8.9-fold higher on Day 14 than on Day 7. Expression on Day 21 was 1.1-fold higher than on Day 7 and lower compared to that on Day 14 (Figure 2(e)).

#### 3.1.2. Regeneration of Dental Pulp and Periodontal Ligament by Heterotopic Root Graft with Dental Pulp Stem Cell-Conditioned Medium

**Figure 3:**
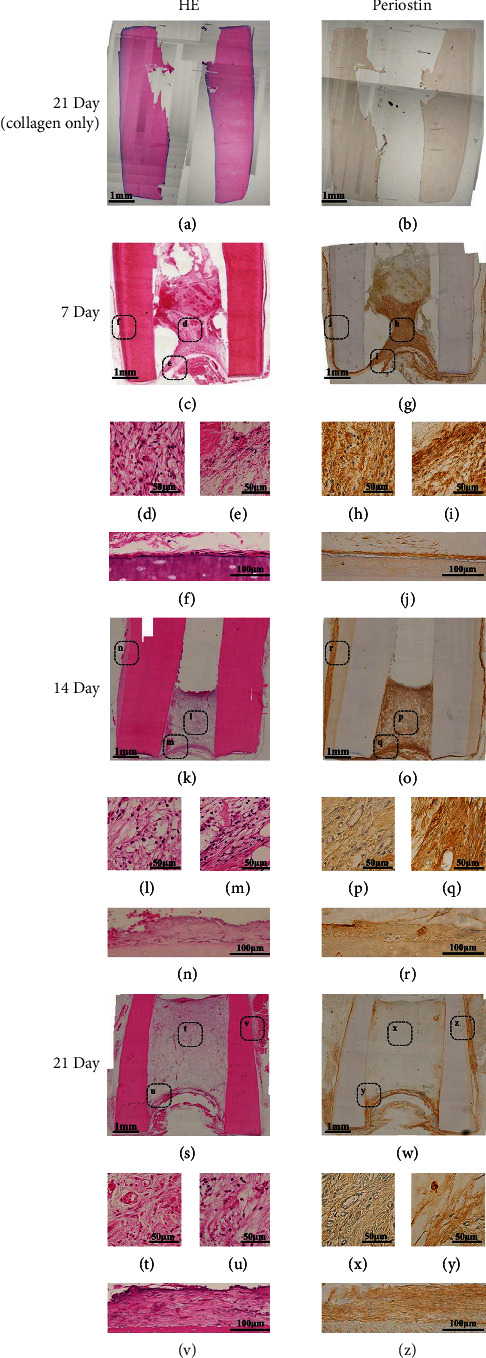
Regeneration of dental pulp and periodontal ligament by heterotopic root graft with dental pulp stem cell-conditioned medium. (a, c–f, k–n, and s–v): HE-stained images. (b, g–j, o–r, and w–z): immunohistochemical staining for periostin. (d, h, l, p, t, and x): central area of the pulp. (e, i, m, q, u, and y): aperture area of the pulp. (f, j, n, r, v, and z): periodontal ligament.

**Figure 4:**
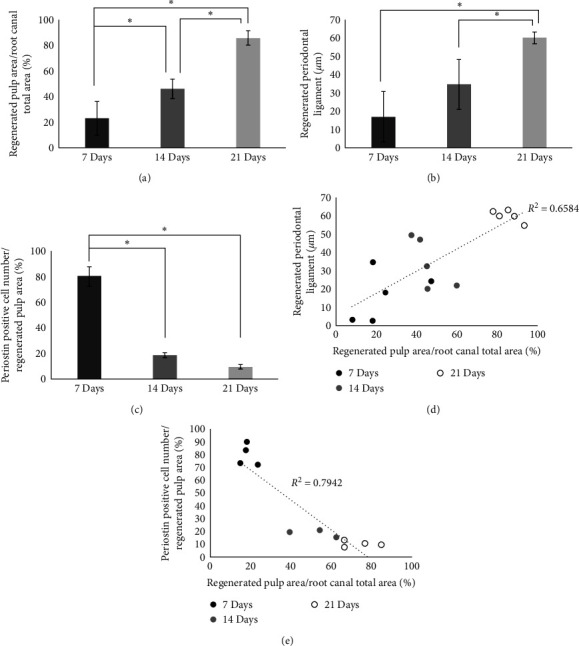
Amount of pulp and periodontal ligament regeneration in regenerated tissue. (a) Amount of pulp regeneration in days. (b) Amount of periodontal ligament regeneration in days. (c) Percentage of Periostin Positive in Regenerated pulp in Days. (d) Correlation between regenerated pulp volume and regenerated periodontal ligament thickness. (e) Correlation between the amount of regenerated pulp and the number of Periostin-positive cells in the regenerated pulp.  ^*∗*^*P* < 0.01.

Type I collagen and CM were mixed and injected into human permanent tooth, and the amount of regenerated tissues inside and outside teeth roots after heterotopic root grafting was analyzed. The ratio of regenerated tissue area to root canal area on HE-stained images (Figure 3) was considered to be regenerated dental pulp volume (Figure 4(a)). No tissue generation was found inside or outside negative-control grafts kept for 21 days after injecting type I collagen (Figures 3(a) and 3(b)). In histological images of the CM graft, the regenerated pulp area was 23% on Day 7. The aperture and central area of the pulp were rich in fibroblasts, but there were no microvascular structures and no odontoblasts in the inner wall of the root canal (Figure 3(c)–3(e)). On Day 14, the regenerated pulp area was 46%, and the aperture and central area of the pulp remained rich in fibroblasts. In addition, microvascular structure had developed (Figure 3(k)–3(m)) and there were odontoblasts in the inner wall of the root canal (Figures 3(b) and 3(e)). On Day 21, the regenerated pulp area had increased to 85%. Tissue images were similar to those on Day 14, showing fibroblast-rich and abundant microvascular structures, as well as continuous circular structures of blood vessels from the aperture to the closure (Figure 3(s)–3(u)). Parallel cell groups were also observed on the inner wall of the root canal, with their cell processes extending into the dentin tubules (Figures 3(c) and 3(f)).

Next, the tissues of the outer surface of the tooth root of the graft were analyzed. Immunohistochemical staining for periostin, a marker of the periodontal ligament, was performed on the external surface of the grafted tooth root. Fibroblasts running horizontally with the tooth root axis and those running vertically and embedded in the tooth root were observed in the tissues on Days 7, 14, and 21 of transplantation (Figures 3(f), 3(j), 3(n), 3(r), and 3(z)). HE staining on Day 21 also showed vascular structures in the periodontal ligament (Figure 3(v)). A 17-*μ*m layer of periostin-positive cells was also observed on Day 7 (Figure 3(j)) and there was no significant difference between this layer and that on Day 14 (Figure 3(r)). On Day 21, the thickness of regenerated periodontal ligaments reached 60 *μ*m (Figure 3(z)), indicating a significant increase compared to Day 14 (Figure 4(b)). Cells were strongly periostin-positive in the aperture and central pulp on Day 7, but only in the aperture of the root on Day 14 (Figures 3(g)–3(i) and 3(o)–3(q)). No strong positivity for periostin was found in the central part of the pulp on Day 14, or in the aperture and central part of the pulp on Day 21 (Figures 3(p), 3(w), 3(x), and 3(y)). There was a positive correlation between the amount of pulp regeneration and the thickness of the regenerated periodontal ligament (Figure 4(d)).

#### 3.1.3. Spatiotemporal Analysis of DSPP, Nestin, and Periostin in Regenerated Dental Pulp Tissues

**Figure 5:**
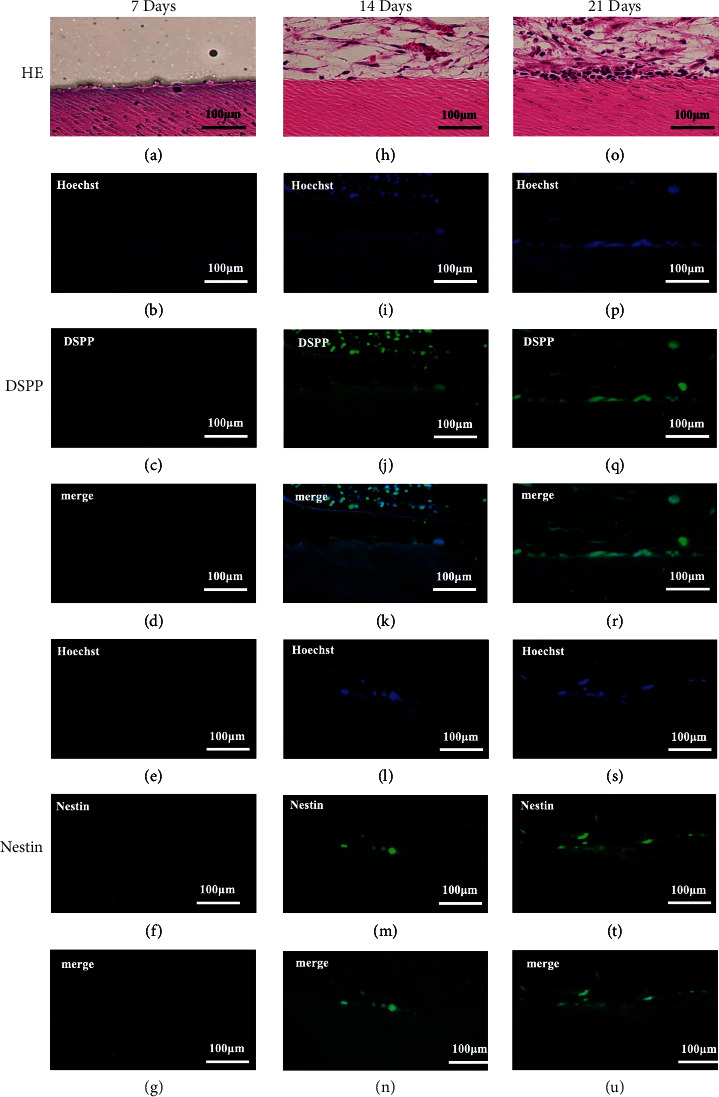
Expression of *DSPP* and *nestin* in regenerated dental pulp tissue. HE stained image of inner wall of regenerated pulp dentin (a, h, and o). Immunohistochemical staining images of inner wall of regenerated pulp dentin. Bule: Hoechst 33342 (b, i, and p), Green: DSPP (c, j, and q), and merge (d, k, and r). Immunohistochemical staining images of inner wall of regenerated pulp dentin. Bule: Hoechst 33342 (e, 1, and s), Green: Nestin (f, m, and t), and merge (g, n, and u).

Regenerated dental pulp after heterotopic root grafting was first immunohistologically stained to analyze localization of DSPP and nestin. On Day 7, there were no parallel cell groups in the inner wall of the root canal and no expression of DSPP (Figure 5(a)–5(g)). Parallel cell groups were observed in the inner wall on Days 14 and 21, with cell processes extended into the dentin tubules. DSPP expression was found in and near the parallel cells. Nestin expression was found in the parallel cells (Figure 5(h)–5(u)). Periostin-positive cells in the regenerated pulp tissue after heterotopic root grafting were measured to estimate the ratio to the regenerated pulp volume. The Periostin positivity on Day 7 (80%) was significantly higher than on Day 14 (19%) and Day 21 (10%) (Figure 4(c)), with no significant difference between Days 14 and 21. There was a negative correlation between the % Periostin positivity and the regenerated pulp volume (Figure 4(e)).

#### 3.1.4. PCR Evaluation of DSPP, Nestin, DMP1, Runx2, and Periostin Expression in Regenerated Dental Pulp Tissues

**Figure 6:**
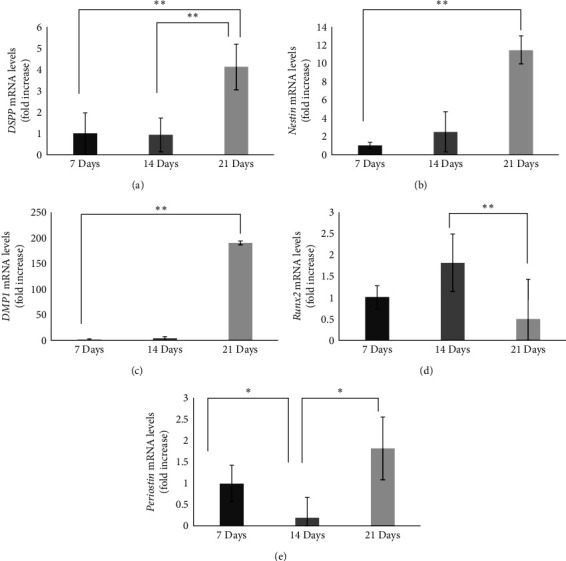
PCR evaluation of *DSPP*, *nestin*, *DMP1*, *Runx2*, and *periostin* expression in regenerated dental pulp tissues Relative gene expression levels 7, 14, and 21 days in regenerated dental pulp tissue. (Relative quantification by *ΔΔCt* method after qPCR analysis; (a) *DSPP*, (b) *nestin*, (c) *DMP1*, (d) *Runx2*, and (e) *periostin*). Mean ± standard error with *n* = 4,  ^*∗*^*P* < 0.05 and  ^*∗∗*^*P* < 0.01.

The expression levels of *DSPP*, *nestin*, *DMP1*, *DMP1*, *and periostin* in the regenerated dental pulp tissues were analyzed by qPCR on Days 7, 14, and 21 after heterotopic root grafting, and the expression dynamics were evaluated. Expression of *DSPP* showed no significant difference between Days 7 and 14, but was significantly higher on Day 7 by 4.1-fold compared to that on Day 21 (Figure 6(a)). Expression of *nestin* on Day 21 was 11.4-fold that on Day 7 (Figure 6(b)). Expression of *DMP1* on Day 21 was 190-fold that on Day 7 (Figure 6(c)). Expression of *Runx2* there was a significant difference between Days 14 and 21, but not between Days 7 and 21 (Figure 6(d)). Expression of *periostin* on Day 14 was 0.2-fold that on Day 7, and that on Day 21 was 1.8-fold that on Day 7. There was a significant difference between Days 14 and 21, but not between Days 7 and 21 (Figure 6(e)).

#### 3.1.5. Promotion of Cell Proliferation by Periostin

**Figure 7:**
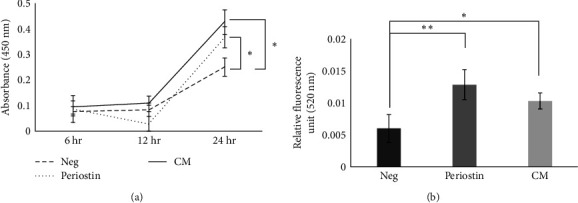
Promotion of cell proliferation and haptotaxis by periostin. (a) Proliferation of dental pulp stem cells. (b) Haptotaxis of dental pulp stem cells in type I collagen.  ^*∗*^*P* < 0.01 and  ^*∗∗*^*P* < 0.05.

Proliferation of dental pulp stem cells was evaluated after addition of periostin and CM. There was no significant difference between the additive and additive-free groups after 6 and 12 hr, but cell proliferation was significantly greater in the additive group at 24 hr. There was no significant difference between the periostin-and CM-treated groups at 24 hr after addition (Figure 7(a)).

#### 3.1.6. Promotion of Cell Haptotaxis by Periostin

Haptotaxis of dental pulp stem cells in type I collagen was evaluated after addition of periostin and CM. There was a significant increase in the periostin-and CM-treated groups compared to the additive-free group at 24 hr after addition. There was no significant difference between the periostin-and CM-treated groups at 24 hr (Figure 7(b)).

## 4. Discussion

Regenerative medicine is currently used in various fields, and its commercialization, industrialization of stem cell processing, and clinical research are in progress [16, 17]. However, the main focus is on use of stem cells, but their amplification, shelf life, and immune rejection limit application to treatment of dental trauma, which is an emergency treatment [18, 19]. Therefore, there is a need to develop regenerative therapy for comprehensive healing of damage caused by trauma in pulp, periodontal tissue, and alveolar bone, as well as in teeth.

Dental roots form and grow while taking in minute amounts of growth factors [20], and release these factors during injury to promote the repair response. Periostin is thought to be such a growth factor [21]. Expression of periostin is markedly upregulated by TGF-*β*1 and BMP-2 [22, 23], and periostin has been identified as an adhesion molecule for preosteoblasts and is involved in osteoblast mobilization, binding, and elongation [9]. Based on findings in periostin-knockout mice, periostin is involved in activation of osteoclasts in alveolar bone around tooth roots, associated bone resorption and root resorption, and local bone metabolism [24]. In addition, periostin is also involved in regulation of calcification in dentin development [25].

When pulp stem cells were cultured in dentin induction medium, *DSPP*, RNA was isolated from the induced cells and analyzed by qPCR. Expression of *DSPP* was significantly higher on Day 21 than on Day 7, with no significant difference between Days 14 and 7. Expression of *nestin*, an odontoblast differentiation marker, and *Runx2*, a bone-related gene, on Day 21 was higher than that on Day 7. The expression of *DMP1*, a secretory activity marker of odontoblasts, did not change with the number of induction days. Therefore, it can be said that odontoblasts were matured by dentin induction. It has been reported that Runx2, a bone-related gene promoter, plays an important role in controlling osteoblast differentiation and maintaining bone homeostasis by binding BET proteins such as BRD4. It has also been reported that differentiation of odontoblasts requires pores with the same diameter as the tubule structure of the dentin wall. In this dentin induction experiment, it is thought that Runx2 was highly expressed because the microenvironment could not be reproduced. Therefore, it is considered necessary to examine dentin differentiation in regenerated dental pulp.

In contrast, *periostin* expression was significantly higher on Day 14 of induction than on Day 7, but there was no significant difference between Days 14 and 21. *Periostin* expression in development and homeostasis of dental roots has been suggested to negatively regulate calcification [25]. Our results suggest that *periostin* expression negatively regulates the odontoblast differentiation process of dental pulp stem cells. Protein expression of any of the factors was confirmed only on Day 21 and was not evaluated over time. This may also be due to the inability to reproduce the microenvironment.

In recent years, tissue regeneration therapies have been developed by reproducing and mimicking changes in the activity of signaling pathways and cell polarity during development and applying them to control the microenvironment [12, 26, 27]. We have shown that dental pulp can be regenerated by transplantation of pulp, bone marrow, or adipose stem cells into the tooth root. In other words, it has been shown that stem cells can regenerate tissues according to the microenvironment growth factor of the transplanted site [28]. We tried to understand the contribution of periostin to the microenvironment through chronological evaluation of periostin dynamics in tissues after transplantation.

It is important to understand and reproduce the microenvironment *in vivo* to develop regenerative therapy as a new trauma treatment, but composite tissue regeneration must take into account the direct involvement of tissues *in vivo*, and this requires both *in vitro* and *in vivo* evaluation systems. Therefore, in the current study, we investigated whether pulp and periodontal ligament regeneration can be produced simultaneously in heterotopic root graft models. Regenerated tissues inside the tooth root showed fibroblasts and regenerated pulp with abundant microvessels. The parallel group of cells in the inner wall of the root canal also expressed *DSPP* and *nestin*, a dentinoblast marker. These findings suggest that the regenerated pulp matured with time after transplantation.

Histological evaluation of neoplastic tissues on the outer surface of the tooth root showed fibroblasts running horizontally with the tooth root axis and running vertically and embedded in the tooth root in the tissues on Days 7, 14, and 21 after transplantation. These tissues also expressed *periostin*, a periodontal ligament-specific marker, which suggests that the adherent tissue was periodontal ligament tissue.

The expression of *periostin* in regenerating tissues was different from *in vitro* results. The dental pulp is surrounded by dentin, which is a hard tissue, and is known to calcify due to aging and a protective response to stimuli [30]. However, it is expected to have a negative control mechanism for calcification because it does not calcify completely and maintains homeostasis. Negative regulation of mineralization by periostin has been reported in odontoblastic cell lines, pulp development, and periodontal ligament. On the other hand, it has been reported that periodontal ligament cells, odontoblasts, and osteoblasts positively regulate the mineralization of periodontal cells [25]. These conflicting results suggest that periostin's action has spatiotemporal specificity. *Runx2* is highly expressed during the differentiation and maturation of mesenchymal stem cells from preosteoblast to immature osteoblast to mature osteoblast. After further differentiation into osteocytos, *Runx2* expression is suppressed when *Dmp1* is expressed. On the other hand, it has been reported that *Runx2* is highly expressed when mesenchymal stem cells differentiate and mature into preodontoblasts, but that *Runx2* expression is suppressed and *DSPP* and *nestin* are highly expressed during maturation to mature odontoblasts via immature odontoblasts [31]. Also in the regenerated dental pulp in this experiment, *Runx2* is highly expressed at Day 14 prior to *nestin*, *DSPP* and *Dmp1*. On day 21, *Runx2* expression was suppressed, and *nestin*, *DSPP*, and *Dmp1* were highly expressed. This is consistent with previous reports, and it is thought that a similar calcification mechanism works in the regenerated dental pulp. In addition, periostin expression increased or decreased with the number of transplant days. At day 7, expression was observed throughout the dental pulp, suggesting that it negatively regulates pulpal mineralization as reported in development. In the regenerated dental pulp, the expression of *periostin* was suppressed 14 days after transplantation, while *DSPP*, *nestin*, and *DMP1* were expressed. Based on this result, since the number of odontoblasts also increases with the increase in the amount of regeneration, the expression level of *periostin* increases prior to the maturation of odontoblasts in the regenerated tissue on day 21. It is considered possible. In addition, *Runx2* expression is suppressed during odontoblast maturation. Therefore, periostin may be involved in the maturation of odontoblasts. Periostin is reported to be involved in angiogenesis and to be a marker for fibrillization. In this experiment, the increased amount of regenerated pulp may have been accompanied by an increase in blood vessels, and pulp maturation and an increase in fiber components may have led to the increase in mRNA. *Periostin* is also expressed in various tissues, including ameloblasts and pulp cells, suggesting that *periostin* may respond to influx of various cells from the host. Overexpression of *Runx2* enhances odontoblast mineralization. On the other hand, periostin suppresses calcification in the dental pulp [25, 32]. In this study, after sufficient pulp regeneration, *Runx2* expression was suppressed and *periostin* expression was enhanced. Therefore, it is inferred that there is a control mechanism of odontoblasts by the interaction of Runx2 and periostin, and it is involved in the maintenance of homeostasis. Overexpression of *Runx2* enhances calcification of dentinoblasts. Periostin, on the other hand, inhibits calcification in the pulp. In the present study, after adequate pulp regeneration, *Runx2* expression was suppressed and periostin expression was enhanced. Therefore, we speculate that there is a regulatory mechanism of dentinoblasts through the interaction of Runx2 and periostin, which is involved in the maintenance of homeostasis.

Periostin also plays an important role in early repair through involvement in migration and differentiation of fibroblasts after acute myocardial infarction [33]. Periostin-positive cells were expressed at sites and times of high proliferation and migration. In mesenchymal stem cells, periostin regulates *CXCL12* expression and has key roles in migration and proliferation [34]. Periostin-positive cells in regenerated dental pulp were observed in the cell influx area and upper part of the regenerated tissue, regardless of the time after transplantation, which suggests that these cells are involved in migration and proliferation. Therefore, we examined proliferation of pulp stem cells and haptotaxis of type I collagen with addition of periostin. The results showed that periostin promoted proliferation of dental pulp stem cells and haptotaxis. In traumatic tooth fracture, vital pulpectomy is now the first-line therapy from the viewpoint of pulp preservation. Vital pulpectomy is curative and is based on a mechanism of migration of pulp stem cells to the amputation site, formation of a dentin bridge, and closure of the wound surface. The main component of the pulp matrix is type I collagen, through which pulp stem cells migrate. Periostin promotes proliferation of pulp stem cells, migration in type I collagen, and calcification in regenerated pulp, and thus, may be an important factor in the microenvironment of regenerated pulp. This suggests the feasibility of development of new trauma treatment using periostin.

## 5. Conclusions

In this study, a heterotopic root graft model showed regeneration of pulp inside the tooth root, periodontal ligament on the outer surface, and tissues corresponding to the surrounding microenvironment. Since these regenerated tissues were observable on the same slice, it was possible to understand changes in the microenvironment within the tissues. Periostin promoted proliferation of pulp stem cells, migration in type I collagen, and calcification in regenerated pulp, which strongly suggests that periostin is a promising candidate as a factor that contributes to the microenvironment of regenerated pulp.

## Figures and Tables

**Table 1:** Human primers for reverse transcription-polymerase chain reaction.

Gene name		5′ ⟵ DNA sequence ⟶ 3′	Amplicon size	Accession number
*DSPP*	Forward	GCCATTCCAGTTCCTCAAAGC	106	NM_006475.3
	Reverse	CATTTAACTCATCCTGTACTGACACA		

*Nestin*	Forward	CCTCAGCTTTCAGGACCCCAAG	139	NM_016701
	Reverse	TGGCACAGGTGTCTCAAGGG		

*DMP1*	Forward	ATCCTGTGTCTCCCAGTAACC	94	NM_004407
	Reverse	GGGTGGTGTTGGTGCCTGA		

*Runx2*	Forward	ATAACCGTCTTCACAAATCCTCCC	100	NM_001024630
	Reverse	GCTTCTGTCTGTGCCTTCTGG		

*Periostin*	Forward	CAAGGGAGAAACGGTGCGATT	144	NM_014208.3
	Reverse	AAGTAGGCTGAGGAAGGTGCTAA		

*β*-*actin*	Forward	GAGCACAGAGCCTCGCCTT	197	NM_001101.5
	Reverse	GCCCACCATCACGCCCTG		

**Table 2:** Murine primers for reverse transcription-polymerase chain reaction.

Gene name		5′ ⟵ DNA sequence ⟶ 3′	Amplicon size	Accession number
*DSPP*	Forward	ACGGTGTTGAAGAAGGCGAC	147	NM_010080.3
	Reverse	CAGTTTCTATTCCCTGATCTTGGC		

*Nestin*	Forward	TGGAAGGTGGGCAGCAACT	78	NM_016701
	Reverse	CCTCCAGCAGAGTCCTGTATGT		

*DMP1*	Forward	GTGTCTCTCCCAGTTGCCAG	116	NM_001359013
	Reverse	CTTTCTTCTGATGACTCACTGTTCGT		

*Runx2*	Forward	ACCACGCCGCTGTCTTCC	87	NM_001146038
	Reverse	GGTGGCAGTGTCATCATCTGAA		

*Periostin*	Forward	AGACGACCTTTCATCATTTAGAGCA	87	MN_001198765
	Reverse	GCAAAGAGCGTGAAGTGACCAT		

*β-actin*	Forward	CACTGTCGAGTCGCGTCCA	79	NM_007393.5
	Reverse	CGAACTGGTGGCGGGTGT		

## Data Availability

The data that support the findings of this study are available upon request from the corresponding author.
